# Bullying Victimization and Trauma

**DOI:** 10.3389/fpsyt.2020.480353

**Published:** 2021-01-14

**Authors:** Thormod Idsoe, Tracy Vaillancourt, Atle Dyregrov, Kristine Amlund Hagen, Terje Ogden, Ane Nærde

**Affiliations:** ^1^Norwegian Center for Child Behavioral Development, Oslo, Norway; ^2^Department of Psychology, University of Ottawa, Ottawa, ON, Canada; ^3^Center for Crisis Psychology, University of Bergen, Bergen, Norway

**Keywords:** bullying victimization, consequences, PTSD (posttraumatic stress disorder), complex PTSD, developmental trauma disorder

## Abstract

Bullying victimization and trauma research traditions operate quite separately. Hence, it is unclear from the literature whether bullying victimization should be considered as a form of interpersonal trauma. We review studies that connect bullying victimization with symptoms of PTSD, and in doing so, demonstrate that a conceptual understanding of the consequences of childhood bullying needs to be framed within a developmental perspective. We discuss two potential diagnoses that ought to be considered in the context of bullying victimization: (1) developmental trauma disorder, which was suggested but not accepted as a new diagnosis in the DSM-5 and (2) complex post-traumatic stress disorder, which has been included in the ICD-11. Our conclusion is that these frameworks capture the complexity of the symptoms associated with bullying victimization better than PTSD. We encourage practitioners to understand how exposure to bullying interacts with development at different ages when addressing the consequences for targets and when designing interventions that account for the duration, intensity, and sequelae of this type of interpersonal trauma.

## Introduction

In this article, we argue that bullying victimization be considered a repetitive interpersonal trauma where reactions are understood within the combined framework of a developmental trauma disorder and a complex post-traumatic stress disorder. This comprehensive and integrated understanding requires that the research gap between the fields of bullying and interpersonal trauma to be bridged.

Even though exposure to bullying is about being harmed intentionally by others, it is unclear from the literature whether bullying victimization (i.e., being the target of bullying; henceforth called bullying) should be considered as a form for interpersonal trauma. With some exceptions, the bullying and trauma research traditions operate quite separately. This is confirmed by examining the table of contents of bullying and trauma journals, as well as conference proceedings and agendas related to conventions within these two respective fields. Trauma journals and trauma conferences seem more or less to lack contributions about the topic of bullying. There may be several reasons why this is the case. Originally, the bullying research tradition among children and adolescents emerged from the educational field where the purpose was to define the phenomenon, estimate prevalence, and understand its etiology (1, 2). The intent was to help identify perpetrators and implement effective interventions to stop this devastating behavior (2). Trauma research, emerged within the fields of psychiatry and psychology (3), with a focus on how to reduce or heal symptoms attributed to traumatic experiences. From its conception, there has been discussions about what constitutes trauma and which criteria must be satisfied in order to define a life event as traumatic (4). This discussion is particularly pertinent for the classification of trauma required for the diagnosis of post-traumatic stress disorder [PTSD; American Psychiatric Association (5)]. Perhaps the focus on the psychiatric sequelae is a contributing factor for why research on bullying has not been integrated within the trauma field. There is also some disagreements about whether bullying can be classified as a traumatic event and whether it can satisfy the diagnostic criteria for PTSD (6).

In this article, we discuss these issues. First, we review studies that connect bullying with symptoms of PTSD. We then demonstrate that the research on the outcomes of bullying show far more complex consequences than the classic symptoms of PTSD. A discussion concerning whether the negative mental health correlates and outcomes of bullying are related to the fact that bullying often goes on over time, sometimes for years, and thus should be approached within a developmental perspective is advanced. Given the stability of bullying victimization (7), we discuss another potential framework that could be better suited for understanding the consequences of bullying. Specifically, the “developmental trauma disorder” (DTD) that was suggested as a new diagnosis for the Diagnostic and Statistical Manual for Mental Disorders 5 [DSM-5, (5); see (8, 9)]. Even though the proposed diagnosis was not accepted by the DSM-5 committee, a somewhat similar diagnosis, termed “complex post-traumatic stress disorder” has been included in the ICD-11 in the new conceptualization of stress-related disorders (10). As the DSM-5 is still the predominant disorder classification system used for research on trauma, we nevertheless discuss whether it could be a source for, or give ideas to, the development of a possible conceptual framework for an integrated understanding of the consequences of being exposed to bullying within a trauma perspective. This idea is explored by examining how empirically established consequences of bullying fit with the symptomatology defined for the proposed diagnostic criteria for DTD. We conclude by discussing the significance this may have for future research and practice.

## Conceptual Definition and Prevalence of Bullying

Bullying is understood as a systematic abuse of power (11–13). An often used operationalization of bullying is provided by Olweus (2) who states that a “A student is being bullied or victimized when he or she is exposed, repeatedly, and over time, to negative actions on the part of one or more other students” (p. 1,173). Olweus further adds that “in order to use the term bullying, there should also be an imbalance in strength” (p. 1,173).

Bullying can also happen to adults (6). Although there seems to be some consensus to define cyberbullying as bullying that occurs via the internet or cell phones, some researchers are more specific in terms of taxonomies and measurement (14).

The prevalence of bullying depends on which population is studied and how bullying is defined and operationalized. For example, child respondents self-report different prevalence rates depending on whether they are provided with a definition of bullying or not in the questionnaire (13). Even when a standardized definition is provided, along with identical measures and sampling procedures, large differences are still noted between countries. For example, Craig et al. (15) report prevalence rates ranging from 5% to about 45% in a cross-national study of bullying conducted in 40 countries. When it comes to gender differences, the meta-analysis by Cook et al. (16) of 153 studies demonstrated that boys were more involved in bullying as perpetrators, targets, and target-perpetrators (i.e., bully-victims, targets who become bullies), although the strength of the gender effect varied somehow for these three groups. In terms of ethnic group difference in bullying victimization, a recent meta-analysis by Vitoroulis and Vaillancourt (17) demonstrated no main effect difference across countries among ethnic majority and minority children and adolescents. However, moderator analyses indicated that ethnic majority youth were more exposed to peer victimization than minority youth in the US (Cohen's *d* = 0.23).

## Bullying and PTSD Symptoms

The major diagnosis that captures the aftermath of potential traumatic experiences is PTSD. In recent years, several studies have revealed strong associations between bullying and PTSD symptoms (18–24). Two studies found that between 30% and 40% of bullied teens scored above the clinical cutoff for PTSD symptoms (20, 25). Rivers (23) investigated 119 individuals who identified as lesbian, gay, or bisexual, and about 25% of them reported having trouble with negative memories of bullying well after leaving school. Seventeen percent had profiles of PTSD in accordance with the DSM-IV (26), with one in 10 reporting that they regularly experience flashbacks. In a recent meta-analysis by Nielsen et al. (6) representing 2,132 children from seven combined studies, a correlation of 0.39 (95% CI: 0.24–0.52; *p* < 0.01) was reported between bullying and an overall score for PTSD symptoms. The authors concluded that there was a strong association between bullying exposure and PTSD symptoms in children and adolescents.

## Is Bullying a Traumatic Event and Thereby Results in a Traumatized Response?

PTSD is different from most other psychiatric disorders insofar as there is an established link between exposure to (a) traumatic event(s) and resulting symptoms (5). Eight diagnostic criteria are listed in the DSM-5 (labeled A through H). Of relevance to our review is the A criterion (stressor) which states that the person was exposed to “actual or threatened death, serious injury, or sexual violence” (p. 271). The exposure can be direct, as a witness, by learning that a relative or close friend was exposed, or indirectly by being exposed to aversive details of the trauma (e.g., as first responders medics). Whether bullying fulfills the A criterion for PTSD depends on how the A criterion is understood and interpreted. Although criterion A refers to exposure to “actual or threatened death, serious injury, or sexual violence,” it is not clear if exposure to bullying satisfies this condition. Idsoe et al.'s (27) review concluded that it remains unclear if criterion A is indeed met. However, two studies involving adults suggested that bullying does fulfill the A criterion for PTSD (28, 29). In a third study, Signorelli et al. (30) concluded that the A criterion was not fulfilled, and thereby PTSD was not regarded as an adequate diagnosis for exposure to bullying. There were several problems with these studies that raise concerns about their conclusions. All three studies had small sample sizes [from *n* = 1 (case study) to *n* = 64], as well as poor descriptions of, and lack of control for, potential alternative traumatic events that could have been present before, during, or after the period that bullying took place. Nielsen et al. (6) pointed to these problems when they concluded that no existing studies could provide good evidence for or against bullying exposure as satisfying the A criterion for adults and recommended that future studies investigate these issues using longitudinal designs and clinical interviews.

What about the studies mentioned above (20, 25) that reported clinically significant levels of PTSD symptoms among bullied children? The problem with these studies is that the symptoms could indicate disorders other than PTSD. Moreover, because we do not have valid information about the duration of symptoms, we cannot disentangle the problems from alternative diagnoses of acute stress disorder (when symptoms consistent with PTSD last for a minimum of 2 days and a maximum of 4 weeks after the traumatic event) or adjustment disorder (when symptoms consistent with PTSD occur in response to a stressor that is not consistent with the A criterion, they start within 3 months after exposure, and resolve within 6 months—if not, they occur in response to a chronic stressor).

There has been a general discussion within the trauma field about whether the A criterion has been given too much importance [e.g., (4)]. Bedard-Gilligan and Zoellner (31) studied this within three different samples: (1) undergraduate women recruited through participant pools at two large metropolitan university campuses, (2) undergraduate men and women recruited through an undergraduate participant pool at a third metropolitan university campus, and (3) women responding to advertisements seeking women with trauma histories. Participants were included if they endorsed an event that “bothers you the most” from a checklist of events (Item 14) on the Post-traumatic Diagnostic Scale [PDS; (32)]. Bedard-Gilligan and Zoellner (31) calculated rates of criterion A events and PTSD and applied them to investigate the predictive utility of Criterion A for PTSD symptoms, duration, and functional impairment. The Criterion A did not predict much better than chance and the authors questioned the importance of this criterion. In another study, Robinson and Larson (33) found that life events like expected death, serious illness of someone close, romantic relationship problems, family relationship problems, predicted similar, if not higher, levels of PTSD symptoms than individuals reporting a traumatic event in accordance with the A criterion. These researchers questioned whether traumatic events are necessary to elicit symptoms of post-traumatic stress. Based on such discussions, the removal of the A criterion from the PTSD diagnosis has been suggested because of the possibilities of identifying people with high levels of symptoms without it (4). It should be noted however, that the DSM-5 committee narrowed the definition of trauma, to be specific to life threatening events or sexual violence. This was done to avoid what some researchers labeled “criterion creep” (34, 35), which refers to expanding the pool of qualifying events. Notwithstanding this important discussion, it is worthy to recognize that studies have identified a wide range of childhood events beyond exposure to bullying that are associated with PTSD symptoms without the stressor meeting the DSM-5 criteria for trauma [see (34), for a review]. It is also possible that bullying sometimes, but not always, constitutes a life event that satisfies the A criterion. There are cases in which children and adolescents feared for their lives because of being bullied by their peers. For example, a former student from Howell Cheney Technical High School in the US sued the state after being bullied during her junior year, claiming that she feared for her life (36). In our clinical encounters we have also had clients describe that they thought they were going to die in relation to being bullying.

## If Bullying is a Traumatic Event for Some, Are Symptoms of PTSD the Major Outcome?

Even though bullying is associated with PTSD symptoms, it has also been linked to a range of other mental health outcomes like loneliness, anxiety, depression, suicide ideation/attempts (37–39). Terr (40, 41) suggested two categories of trauma—Type I traumas and Type II traumas—that may elicit different reactions. Type I trauma is mainly the result of a single traumatic event like a car accident or an attack by a violent dog, while Type II trauma is the experience of repeated exposure to traumatic situations, like bullying, which by definition, is repeated in nature (11–13). Terr suggested that although Type I traumas are more closely linked to PTSD than Type II traumas, Type II traumas nevertheless seem to result in a much more complex symptomatology. When Bremner (42) suggested the concept “trauma-spectrum disorders,” his reasoning was to capture a whole range of psychological problems associated with childhood trauma, not only PTSD, but also borderline personality disorder, dissociative identity disorder, depression, substance abuse, and psychosomatic problems. Although his proposal was related to child abuse, consequences of bullying, as an interpersonal repetitive trauma, could be conceptualized to capture a range of problems as well. This would be consistent with empirical findings demonstrating complex mental health problems linked to bullying (37–39, 43).

To move forward, we believe a closer look at what is occurring within the field of childhood trauma in general is needed. It has been suggested that early interpersonal childhood trauma like physical or sexual abuse and neglect are associated with complex symptomatology (8, 44). Also questioned is whether traditional psychiatric disorders and possible comorbid diagnoses fit with such complex symptoms and whether assigning traditional diagnoses for such problems could reduce the possibilities of providing proper treatment (8, 9). In 2009, a group of merited researchers within the trauma field proposed to include a diagnosis for children and adolescents in the forthcoming DSM-5 (9) that they named “Developmental Trauma Disorder” (DTD). The authors underscored the importance of understanding childhood trauma within a developmental psychopathological framework (45) with increased attention paid to the effects of early adverse life experiences on brain development (46) in order to establish interventions that were developmentally appropriate. They pointed to the adverse problems that can emerge if children are exposed to chronic interpersonal stressors, especially if they are followed by inadequate caregiving systems from parents, and how these environmental risks could be the onset of developmental trajectories that include a range of emotional and behavioral difficulties. Even if children with complex trauma-related clinical presentations also met diagnostic criteria for PTSD, this diagnosis alone could fail to capture the broader range of psychiatric symptoms that could result in the provision of incomplete or inadequate interventions. van Der Kolk et al. (47) have demonstrated that DTD cannot be reduced to a combination of PTSD plus psychiatric comorbidities nor is it simply a variant of PTSD. They have also argued that evidence-based treatments for PTSD do not address the developmental impairments that many of these children suffer from, even though they may lead to a reduction in PTSD symptoms.

In the literature on developmental trauma, little is explicitly stated about bullying, although it is sometimes briefly mentioned like when D'andrea et al. (48) mentioned that “victimization in childhood may take many forms, including assault, abduction, bullying, and neglect” (p. 187–188). DTD was suggested based on studies of child sexual abuse and exposure to family violence. Exposure to these forms of interpersonal violence is often part of children's life from very early on and can impact the development of the brain's anatomy, functionality, and connectivity [e.g., (49); see review by (50)]. When applying a developmental perspective, we need to understand how exposure interacts with development at different ages (e.g., pre-adolescence vs. adolescence). For example, will a child with a “safe” early childhood be less susceptible to later stress? [For a review of PTSD and the neurodevelopmental network perspective, see (51)].

Even though bullying exposure does not fulfill the kind of event(s) needed to satisfy the proposed diagnosis of DTD, it is useful to examine empirical findings to see how the sequelae of symptoms fit with the suggested criteria for DTD proposed for the DSM-5. Toward this aim, we go through the seven main diagnostic criteria (labeled A through F) as proposed by van der Kolk et al. [(9), p. 5–7], and we link each criterion to empirical findings for bullying.

## Proposed Criteria for a Developmental Trauma Diagnosis and How Symptoms Among Children Who Experienced Bullying Fit Within This Framework

### A. Exposure

The child or adolescent has experienced or witnessed multiple or prolonged adverse events over a period of at least 1 year beginning in childhood or early adolescence, including:

A. 1. Direct experience or witnessing of repeated and severe episodes of interpersonal violence; and
A. 2. Significant disruptions of protective caregiving as the result of repeated changes in primary caregiver; repeated separation from the primary caregiver; or exposure to severe and persistent emotional abuse [(9), p. 5].

“Criterion A requires multiple, ongoing exposures to both interpersonal violence and disruptions in caregiving” [(9), p. 8].

#### Criterion A and Bullying

The nature of bullying exposure fits well with the ongoing negative interpersonal acts that constitutes the A1 criterion. When it comes to the A2 criterion it becomes less clear to what extent this holds true for bullying. Nevertheless, we explore why adequate care/support is regarded important for normal functioning and development. Findings from seminal research in developmental psychology clearly demonstrate that experiencing comforting, responsive, and supportive relationships with secure and predictable primary caregivers are important for adequate development and adaptation (52–55). A fundamental tenet of attachment theory is that the attachment style developed between the infant and the caregiver influences future relationships (56). Indeed, attachment theory has been suggested as a useful conceptual framework for linking problematic parent-child relationships to peer bullying (57) and empirical evidence show that children with secure attachments to parents and peers are less likely to be perpetrators or targets of bullying (58). Moreover, warm, supportive, and well-structured families help protect children from the negative outcomes associated with bullying victimization and thus promote emotional and behavioral resilience to bullying (59). The capacities associated with the regulation of emotions are likely anchored in the nature of the attachment between the child and the primary caregiver in the first year of life. The quality of the responses elicited in the caregiver to meet physiological needs provide the child with a sense of security that consequently is “encoded physiologically in the experience of non-disruptive and need-satisfying regulation of early states” [(60), p. 20].

When a child experiences danger, he/she is likely to experience fear. In this case, caregivers can provide support by functioning as an external regulatory system through soothing, caressing, or talking to calm to the child. Early experiences of such emotion regulating relationships lay an important basis for the child's development of regulatory capacities as a gradual shift from other-regulated to self-regulated affective responses throughout childhood. Children who experience trauma in the context of caregivers that do not provide this kind of support can develop emotion dysregulation that can give rise to adverse psychological symptoms. However, traumas are major developmental events potentially leading to emotion dysregulation even in favorable relationships in the family. Likewise, ongoing unfavorable relationships in the family can cause emotion dysregulation without necessitating the development of DTD. The two problems do not have to occur simultaneously. Many factors can lead to emotion dysregulation in a child and increase the risk of bullying becoming a traumatic event (criterion A1) without implicating criterion A2 (for example genetic, constitutional, and temperamental vulnerabilities—i.e., dysregulated parents having dysregulated children because of the aforementioned factors).

One reason for why children who live in unsupportive, neglectful, or dangerous families show problems with emotion-based regulation is that they can be inclined to be vigilant, distrustful, and wary or they may develop an aggressive and confrontational stance (as modeled at home). Children living in such environments can experience an exaggerated need to defend themselves (61). Some can even act on impulse as a self-protecting strategy in violent homes. The taxing effect of maltreatment or other catastrophic stressors on children may exhaust their socio-emotional resources leaving them less able to integrate external stimuli and their own affective experiences to produce a desired outcome. An additional explanation for their dysregulation may be that they have not had enough experience with “desirable outcomes.” Successful regulation of emotion is at least partly influenced by the goals a child wants to achieve and if these goals are inappropriate, the expression of emotion is likely to be dysfunctional as well.

When children become older, support and care within other social environments becomes important such as relationships with peers, teachers, and coaches (62, 63). Lereya et al. (64) found that children who were bullied by peers only, were more likely than children who were maltreated only [assessed as physical, emotional, or sexual abuse, or severe maladaptive parenting (or both)] to have mental health problems in young adulthood. It is possible that the stronger association found for bullying was because its assessment occurred closer to the onset of mental health problems than the assessment of child maltreatment. Another reason could be that associations with specific abuse types were obscured in the overall maltreatment variable. However, it has also been found that social support moderates the effects of bullying on anxiety/depression (65). These findings suggest that relationships with peers can be associated with a potential “double” negative impact, similar to what is seen with parents. By “double” we mean that peers not only cause pain through bullying, but also fail to provide the type of support needed to cope with the abuse at hand. It is possible that inadequate support from peers and negative acts from peers happen concomitantly, and thereby—when experienced over time—interfere with healthy development. Accordingly, bullying could be interpreted in line with the A–criteria (1 and 2), constituting a kind of developmental trauma by being exposed to long-lasting stress in combination with inadequate support for regulating negative emotions, that again could be reflected in a dysregulated neurobiological stress response system and an under stimulated regulatory system (50, 66). This is in accordance with Harris' group socialization theory (63), postulating that as children get older, the peer group becomes their primary socializing agent, and if this socializing context is problematic, like the socializing context of parents, negative outcomes should be expected. Keeping in mind this new socializing context, it is clear how the A2 criterion can relate to bullying exposure.

### B. Affective and Physiological Dysregulation

The child exhibits impaired normative developmental competencies related to arousal regulation, including at least two of the following:

B. 1. Inability to modulate, tolerate, or recover from extreme affect states (e.g., fear, anger, shame), including prolonged and extreme tantrums, or immobilization.
B. 2. Disturbances in regulation in bodily functions (e.g., persistent disturbances in sleeping, eating, and elimination; over-reactivity or under-reactivity to touch and sounds; disorganization during routine transitions).
B. 3. Diminished awareness/dissociation of sensations, emotions, and bodily states.
B. 4. Impaired capacity to describe emotions or bodily states [(9), p. 5].

#### Criterion B and Bullying

##### B. 1. Inability to Modulate, Tolerate, or Recover From Extreme Affect States

Several studies have shown that children and adolescents who are targets of bullying score particularly high on emotion dysregulation and suppression, reactive aggression, hostility, sadness, and depressive symptoms (67–73).

##### B. 2. Disturbances in Regulation in Bodily Functions

Children/adolescents exposed to bullying are found to be at increased risk for disordered eating behavior (70, 74–76). We recommend that bullying should be considered when evaluating risk and treatment planning for children with eating problems. Targets of bullying and bully-victims also report sleep disturbances (77, 78) and it is therefore recommended to consider sleep problems as a possible sign that a child is being bullied.

##### B. 3. Diminished Awareness/Dissociation of Sensations, Emotions, and Bodily States

There is a significant emerging literature demonstrating that bullying is related to dissociation (79, 80). In a meta-analysis and review of 10 prospective studies, Cunningham et al. (81) found that exposure to bullying prior to age 18 predicted the later development of psychotic symptoms.

##### B.4. Impaired Capacity to Describe Emotions or Bodily States

To the best of our knowledge, there are not many studies directly examining impaired capacity to describe emotions or bodily states in relation to bullying. However, as we have reported, impaired emotional regulation has been noticed (82, 83). In these studies, emotional constriction following parental maltreatment and other emotional regulation problems were risk factors for bullying. Impaired capacity to describe emotions or bodily states are also close to alexithymia, which has been related to bullying (19).

In sum, several studies have established a link between exposure to bullying and several outcomes belonging to the B criteria in the proposed diagnosis of DTD.

### C. Attentional and Behavioral Dysregulation

The child exhibits impaired normative developmental competencies related to sustained attention, learning, or coping with stress, including at least three of the following:

C. 1. Preoccupation with threat, or impaired capacity to perceive threat, including misreading of safety and danger cues.
C. 2. Impaired capacity for self-protection, including extreme risk-taking or thrill-seeking.
C. 3. Maladaptive attempts at self-soothing (e.g., rocking and other rhythmical movements, compulsive masturbation).
C. 4. Habitual (intentional or automatic) or reactive self-harm.
C. 5. Inability to initiate or sustain goal-directed behavior [(9), p. 5].

#### Criterion C and Bullying

##### C. 1. Preoccupation With Threat, or Impaired Capacity to Perceive Threat, Including Misreading of Safety and Danger Cues

Several studies on bullied adolescents have documented important findings with respect to criterion C1—preoccupation with threat, or impaired capacity to perceive threat, including misreading of safety and danger cues, including hostile attributions (84), distressing paranoid thinking and subsequent misappraisal of threat (85), and biased interpretations of social situations and the intentions of others (86). fMRI studies have found that peer victimization is associated with increased neural response to being socially excluded (87–89), greater activation than controls in the amygdala, orbitofrontal cortex, and ventrolateral prefrontal cortex when viewing video clips of facial expressions that depicted negative interpersonal feedback (90), and thicker cortex in the fusiform gyrus compared to children who were not bullied by their peers [see review by Vaillancourt and Palamarchuk (50)].

##### C. 2. Impaired Capacity for Self-Protection, Including Extreme Risk-Taking or Thrill-Seeking

Bullying victimization is associated with several types of health risk behavior such as violence, obesity, decreased physical activity, sexual risk, and substance use (91–94).

##### C. 3. Maladaptive Attempts at Self-Soothing

Criterion C.3 has been documented as chronic masturbation, rocking, self-harm, or other repetitive self-stimulating types of behavior. Bullying victimization is associated with impaired capacity for self-protection, including sexual risk, and substance use (91, 94).

##### C. 4. Habitual (Intentional or Automatic) or Reactive Self-Harm

Exposure to bullying has been related to non-suicidal self-harm such as cutting, self-hitting, skin picking, head banging, and self-burning (95, 96), and suicidal thoughts and attempts (97–99).

##### C. 5. Inability to Initiate or Sustain Goal-Directed Behavior

An inability to sustain goal-directed behavior may include lack of curiosity, difficulties with planning or completing tasks, and/or avolition. Carroll et al. (100) found that twins (*M* = 15.39 years, *SD* = 1.74) who experienced more severe bullying were biased toward detecting goal relevant stimuli during an affective go/no go task. We believe preoccupation with detecting threats triggered by bullying may interfere with and redirect attention from other goal-directed behavior. In a study of 390 African American and Iraqi refugee adolescents, Kira et al. (101) reported that exposure to bullying had significant effects on perceptual reasoning, processing speed, and working memory, after controlling for cumulative trauma and discrimination. Vaillancourt et al. (102) found that peer victimization predicted memory problems over a 2-year period in a study of 168 children, controlling for prior peer victimization, symptoms of depression, and levels of cortisol. These neurocognitive deficits likely interfere with the initiation and sustainment of goal-directed behavior.

The link between being the target of bullying and concurrent and subsequent depression is one of the most robust findings in the literature [see meta-analyses by Moore et al. (94, 103–105)]. Although not directly examined, depression, a disorder of motivation (5), likely interferes with the ability to initiate and sustain goal directed behavior in children who were bullied.

### D. Self and Relational Dysregulation

The child exhibits impaired normative developmental competencies in his/her sense of personal identity and involvement in relationships, including at least three of the following:

D. 1. Intense preoccupation with safety of the caregiver or other loved ones (including precocious caregiving) or difficulty tolerating reunion with them after separation.
D. 2. Persistent negative sense of self, including self-loathing, helplessness, worthlessness, ineffectiveness, or defectiveness.
D. 3. Extreme and persistent distrust, defiance or lack of reciprocal behavior in close relationships with adults or peers.
D. 4. Reactive physical or verbal aggression toward peers, caregivers, or other adults.
D. 5. Inappropriate (excessive or promiscuous) attempts to get intimate contact (including but not limited to sexual or physical intimacy) or excessive reliance on peers or adults for safety and reassurance.
D. 6. Impaired capacity to regulate empathic arousal as evidenced by lack of empathy for, or intolerance of, expressions of distress of others, or excessive responsiveness to the distress of others [(9), p. 6].

#### Criterion D and Bullying

##### D. 1. Intense Preoccupation With Safety of the Caregiver or Other Loved Ones (Including Precocious Caregiving) or Difficulty Tolerating Reunion With Them After Separation

We could not find any studies linking this criterion to bullying exposure, so this remains to be investigated (many of the studies for the DTD proposal involved younger children who are dependent on caregivers).

##### D. 2. Persistent Negative Sense of Self, Including Self-Loathing, Helplessness, Worthlessness, Ineffectiveness, or Defectiveness

Being exposed to bullying is associated with lower self-esteem and poorer self-concept (14, 103), that are more pronounced in children than in adolescents (106). It is noteworthy, however, that most of the studies' participants were between the ages of 8 and 13 years. The development of self-esteem has been shown to be highest at around ages 9–12 and to decrease thereafter (107, 108). Saint-Georges and Vaillancourt (109) found evidence for a self-perception driven model that was characterized by the indirect effect of self-esteem on later peer victimization via depressive symptoms in adolescents followed prospectively for 5 years. Bullying exposure has also been associated with helplessness (110) and shame (71).

##### D. 3. Extreme and Persistent Distrust, Defiance, or Lack of Reciprocal Behavior in Close Relationships With Adults or Peers

Exposure to bullying is associated with distrust of adults (111), paranoid ideation and suspiciousness (112), and psychotic symptoms (81, 113). However, causality needs to be discussed, at least for paranoid thinking and psychotic symptomatology. In contrast to the assumed role of bullying as an environmental trigger, the results of a study conducted by Shakoor et al. (114) suggest that exposure to bullying is linked to self-rated paranoia almost entirely via genetic influences.

##### D. 4. Reactive Physical or Verbal Aggression Toward Peers, Caregivers, or Other Adults

This criterion refers to aggressive behavior which is reactive (i.e., impulsive or dysregulated) as opposed to instrumental (i.e., intentionally coercive or manipulative). A consistent finding from the bullying field is that targets score higher than control children on reactive aggression (115) but this can be moderated by gender and age (12). Haltigan and Vaillancourt (116) found, in a longitudinal study of bullied children, strong associations between child reported reactive temperament and elevated features of borderline personality disorder (BPD). Specifically, being in a high trajectory group membership for elevated BPD features was 10.23 times higher among children bullied by their peers.

##### D. 5. Inappropriate (Excessive or Promiscuous) Attempts to Get Intimate Contact (Including but Not Limited to Sexual or Physical Intimacy) or Excessive Reliance on Peers or Adults for Safety and Reassurance

This criterion refers to inappropriate boundaries often displayed in children exposed to DTD Criteria A traumatic stressors. This may include sexualized behavior, inappropriate physical boundaries, or excessive self-disclosure. It should be kept in mind that with DTD, most of the research is from the sexual abuse field, where intimacy boundaries have been extensively violated. Accordingly, it is expected that this criterion will not be as prominent in relation to bullying exposure. Nevertheless, there are studies showing links between bullying perpetration and increased sexual behavior [i.e., number of partners, younger sexual debut; e.g., (91, 117)], which is linked to higher social status (118). It seems reasonable to assume that some bullied children and adolescents will engage in sexual behavior as a way of elevating their standing in the peer group or to create protective alliances.

##### D. 6. Impaired Capacity to Regulate Empathic Arousal as Evidenced by Lack of Empathy for, or Intolerance of, Expressions of Distress of Others, or Excessive Responsiveness to the Distress of Others

Criterion D.6 refers to an inability to appropriately gauge perspective in social situations, such that one is either excessively responsive to others' emotions, or unable to feel empathy. Such emotional lability can be seen in the features of borderline personality, which has been linked to bullying (116, 119, 120). The link between empathy and bullying is mixed. A review by Van Noorden et al. (121) found an association between lower perspective taking and bullying victimization, whereas others have found non-significant effects (122, 123). Estévez et al. (124) found that targets of school violence scored significantly higher on the dimension of emotional attention, but significantly lower on emotional clarity (more confused about their emotions), and their ability to regulate their emotion, as well as less affective empathy, indicating that they were less able to share the positive emotions of others.

### E. Post-traumatic Spectrum Symptoms

The child exhibits at least one symptom in at least two of the three PTSD symptom clusters B–D [(9), p. 6].

#### Criterion E and Bullying

We refer to our previous section on PTSD symptoms in this article which demonstrates a link between exposure to bullying and PTSD symptoms.

### F. Duration of Disturbance

Symptoms in DTD Criteria B–E at least 6 months [(9), p. 6].

#### Criterion F and Bullying

We did not find any published study explicitly looking into the duration of symptoms in criteria B–E. However, in general we know that consequences of bullying can last for a very long time. Sigurdson et al. (125) found that being involved in bullying at the age of 14–15 years, predicted lower education, increased risk of poor general health, illegal drug use, and poorer spouse/partner relations at the age of around 27. The negative long-term impact of bullying has also been shown in other studies spanning decades post-exposure (64, 126).

### G. Functional Impairment

The disturbance causes clinically significant distress or impairment in at two of the following areas of functioning:

- Scholastic: under-performance, non-attendance, disciplinary problems, drop-out, failure to complete degree/credential(s), conflict with school personnel, learning disabilities or intellectual impairment that cannot be accounted for by neurological or other factors.
- Familial: conflict, avoidance/passivity, running away, detachment and surrogate replacements, attempts to physically, or emotionally hurt family members, non-fulfillment of responsibilities within the family.
- Peer Group: isolation, deviant affiliations, persistent physical or emotional conflict, avoidance/passivity, involvement in violence or unsafe acts, age-inappropriate affiliations, or style of interaction.
- Legal: arrests/recidivism, detention, convictions, incarceration, violation of probation or other court orders, increasingly severe offenses, crimes against other persons, disregard or contempt for the law or for conventional moral standards.
- Health: physical illness or problems that cannot be fully accounted for physical injury or degeneration, involving the digestive, neurological (including conversion symptoms and analgesia), sexual, immune, cardiopulmonary, proprioceptive, or sensory systems, or severe headaches (including migraine) or chronic pain or fatigue.
- Vocational (*for youth involved in, seeking or referred for employment, volunteer work or job training*): disinterest in work/vocation, inability to get or keep jobs, persistent conflict with co-workers or supervisors, under-employment in relation to abilities, failure to achieve expectable advancements [(9), p. 6–7].

#### Criterion G and bullying

Bullying was found to be associated with lower academic achievement (127), poorer health outcomes (125), difficulties in keeping jobs (128), unemployment (126, 129), problems with making or keeping friends (128), and lack of having a romantic partner (126).

## Complex Trauma in ICD-11

In ICD-11, the description of complex trauma is as follows: “Complex post-traumatic stress disorder” (Complex PTSD) is a disorder that may develop following exposure to an event or series of events of an extremely threatening or horrific nature, most commonly prolonged or repetitive events from which escape is difficult or impossible (e.g., torture, slavery, genocide campaigns, prolonged domestic violence, repeated childhood sexual, or physical abuse). All diagnostic requirements for PTSD are met. In addition, Complex PTSD is characterized by severe and persistent (1) problems in affect regulation; (2) beliefs about oneself as diminished, defeated, or worthless, accompanied by feelings of shame, guilt, or failure related to the traumatic event; and (3) difficulties in sustaining relationships and in feeling close to others. These symptoms cause significant impairment in personal, family, social, educational, occupational, or other important areas of functioning (10).

The studies pertaining to bullying and the DSM are relevant for ICD-11 as well. The diagnosis requires an event or series of events of an extremely threatening or horrific nature, where the possibility of escape is difficult or impossible. In our clinical encounters, targets of bullying have described that they thought they were going to die. As for the accompanying overwhelming emotions to intrusive memories such as fear or horror, we expect that reactions can become blunted when targets suppress, blunt, or dissociate over time to escape the emotional pain involved. Memories and associated emotions change over time and individual adaptations take place to accommodate and dampen them. We argue that studies on bullying confirm the subjective experience of this as extremely threatening and that the narrowing or widening of the stressor criterion [Criterion A, see (4)] would make little difference as to whether bullying is a stress related disorder.

Danzi and La Greca (130, 131) have shown that ICD-11 identifies more children with PTSD than DSM-5. However, the DSM systems identified children with complex symptom presentations with non-core symptoms, while ICD-11 identifies children with more severe core PTSD symptoms. It is evident that inclusion of stressors and different symptoms can impact the rates of PTSD and complex PTSD that will be reported in future studies. Although the ICD-11 tries to reduce the number of PTSD symptoms to a smaller number of core elements to ease diagnosis and reduce comorbidity, the DSM-5 has added to the number of symptoms. The DSM-5 definition of PTSD places it somewhere between ICD-11's PTSD and Complex-PTSD definitions (132).

## Neurobiology

From the empirical studies we have reviewed above, it follows that the consequences of bullying exposure in childhood and adolescence are characterized by complexity, revealing a symptomatology that fits with stress-related illnesses (43). It is thereby important to review studies that have linked changes in stress hormones and brain activity to bullying exposure to see whether this is in accordance with the complex sequelae of psychological and functional consequences that can occur in the aftermath of bullying within a conceptual framework of developmental interpersonal trauma. Exposure to child abuse has been related to dysregulation of the hypothalamic-pituitary-adrenal (HPA) axis, suggesting this as an important factor in the development of stress-related disorders caused by interpersonal trauma (133). In this literature, child maltreatment has been linked to both high and low cortisol levels, although more typically, lower levels of cortisol [see meta-analysis by Bernard et al. (134)]. Relatedly, exposure to bullying has also been associated with alterations in the HPA axis [see review by Vaillancourt and Palamarchuk (50)], particularly decreased levels (135–138).

These findings may suggest that long periods of higher cortisol levels caused by a hyperactive HPA axis responding to stress can be followed by hyposecretion as part of an adaptive process (139). However, peer rejection (140) and bullying victimization (102) have also been related to higher levels of cortisol. Vaillancourt et al. (138) found that sex to moderate the association between bullying exposure and cortisol secretion in that for boys, occasional exposure to bullying was associated with higher levels of cortisol, while for girls it was associated with lower levels. Although Vaillancourt et al. (138) interpreted the sex differences to the possibility of higher social goals among girls—having a higher stress perception in relation to being bullied, they also suggest that more severe or chronic stress can result in hyposecretion when compared to occasional stress. These findings underscore that the association between dysregulations of the HPA axis and stress exposure is a complicated process that requires more research (138, 141). For example, in a recent study of preschool aged children, Vaillancourt et al. (142) found an intricate interplay between the social environment and the biobehavioral system of children, suggesting differential susceptibility is at play. Specifically, they found that for boys and not girls, higher levels of bullying victimization was associated with higher levels of physical aggression at lower levels of basal cortisol, while at higher levels of basal cortisol, higher bullying victimization was associated with lower levels of physical aggression. The results were the reverse at lower levels of bullying.

We also remind readers about the findings related to criterion C1 where fMRI studies demonstrated associations with increased neural responses to being socially excluded (87–89), and greater activation than controls in the amygdala, orbitofrontal cortex, and ventrolateral prefrontal cortex when viewing video clips of facial expressions that depicting negative interpersonal feedback.

## Causality, Multiple Victimization, and Developmental Psychopathology

Although the central question for the present review is whether bullying exposure in childhood and adolescence can be interpreted as an interpersonal trauma resulting in complex symptomatology as conceptualized in the proposed diagnosis of DTD (9), we cannot ignore empirical findings showing that exposure to one type of interpersonal trauma increases the risk of exposure to other kinds of victimization (143). Victimization is not randomly distributed, and can for some children, result in what is called “polyvictimization” (144, 145). Finkelhor et al. (144) defined polyvictimization as having experienced multiple types of victimizations, such as exposure to family violence, physical abuse, sexual abuse, and bullying. They found that polyvictims scored higher than one standard deviation above targets of a single type of abuse even though the exposure was chronic/repeated over time (143, 144). For example two longitudinal studies carried out on the UK (144) and USA (64) found that 7% (UK) and 10% (USA) of the children studied were exposed to childhood maltreatment by a caregiver and bullying by peers. In both studies, maltreated children were more likely to be bullied by their peers than children who were not maltreated. The researchers suggested that polyvictimization could signal a more generalized vulnerability for cumulative victimization exposure and that this underscored the need for studies of bullying exposure to assess a broader range of victimization experiences. Because polyvictimization has a high degree of stability (144), the need to investigate bullying exposure within a developmental perspective is further emphasized. Shields and Cicchetti (83) found that early maltreatment within the family increased the chance of being exposed to subsequent bullying victimization. They related this to evidence describing targets of bullying as more aroused and anxious than non-abused children. They suggested that emotion dysregulation as a result of early maltreatment puts children at risk for subsequent bullying. This cumulative process could be understood as a mechanism for polyvictimization and is in line with developmental perspectives suggesting early poor caregiving experiences as causes of later negative peer interactions.

For some children and adolescents, there is stability in victimization through the accumulation of exposures across time; however, longitudinal research suggests that the experience of being bullied does not result in the same symptom pattern over time. Rather, there is marked variability in terms of outcomes [i.e., multi finality; (43, 133, 146)]. It is not clear why exposure to bullying has more impact on some children than others. So far the focus has been mostly on environmental characteristics like family and school. For instance, Bowes et al. (59) found that warm family relationships (i.e., maternal warmth, sibling warmth) and positive home environments helped buffer children from the negative outcomes associated with being bullied. Other studies suggest that genetic mechanisms can moderate the associations between bullying exposure and health outcomes (66, 146). As stated in a report published by the American Academy of Pediatrics “many adult diseases should be viewed as developmental disorders that begin early in life and that persistent health disparities associated with poverty, discrimination, or maltreatment could be reduced by the alleviation of toxic stress in childhood” [(147), p. 323].

## Concluding Remarks—Can the Conceptual Framework of Developmental Trauma Disorder Be Applied to Bullying?

We agree that the provisional cluster for DTD as a construct adds to PTSD. One, because of the specifications in the criterion A, where DTD captures the ongoing nature of many traumatic experiences and includes interpersonal violence and the concomitant disruptions in caregiving. Two, because of the consequences that are characterized by an array of symptoms that are much more comprehensive than the clusters of PTSD. van Der Kolk et al. (47) demonstrated that DTD cannot simply be seen as a variant of PTSD nor just a combination of PTSD plus psychiatric comorbidities. Rather, these authors have argued that the evidence-based treatments for PTSD do not address the developmental impairments that many of these children suffer from, even though they may lead to a reduction in PTSD symptoms.

From our review of the literature it is clear that exposure to bullying is associated with a more complex sequelae than what is captured in traditional PTSD criteria. We suggest that DTD, proposed but not included in the DSM-5, and complex PTSD (ICD-11), capture the symptoms' complexity to a better extent than the DSM-5 PTSD criteria. However, we remind readers that our review is far from exhaustive. Even though DTD provides a better understanding of the consequences of bullying, it still does not represent a complete understanding. Not all the criteria proposed for DTD are linked to bullying and we are unsure whether they would be even if empirical studies existed. DTD was suggested based on studies of child sexual abuse and exposure to family violence that often take part in children's life from very early on. Bullying tends to occur more frequently at a later age (16), and when perpetrated by peers, does not implicate major attachment figures. Still, it is important to understand how exposure to interpersonal traumas interact with development at different ages when addressing consequences and designing interventions. We hope our work will inspire further investigations into the complexities of the consequences of bullying exposure within frameworks like DTD and complex PTSD.

## Limitations

This is not a systematic review and/or meta-analysis but rather a theoretical and conceptual review.

### Implications for Research and Practice

Before we talk about directions related to the consequences of bullying, we think it is important to make it clear that it is crucial that the bullying stops. There will be no effective treatment for those who are bullied until the exposure has come to an end. Effective strategies to stop bullying has been implemented in anti-bullying programs and is described elsewhere for those interested (2, 11).

The complexity and severity of the consequences following bullying are likely related to the intensity and duration of the exposure that interact with a range of risk and protective factors. Treatment of bullied children within the conceptual framework of DTD should acknowledge the importance of a dysregulated stress-response system and problems related to emotion regulation. A first step should be to help children feel safe and support them in how to regulate their arousal (27, 44). A core issue in the treatment of developmental trauma is the focus on how to change the environments from fear-inducing relationships with others into safe environments for healthy development. For treatment, we recommend that a thorough mapping/assessment of the potential traumatic relationships the child has experienced be conducted (criteria A1 and A2), along with the ongoing stressors they face, and the broad array of potential moderators present (e.g., age, gender, genetic vulnerabilities, and access to social support). Assessment must reflect the complexity of interacting factors, as they can contribute to potential multi finality (diversity) of developmental outcomes (148). The treatment should also be tailored to the specific child. After stopping the bullying, increasing the number of healthy relationships is helpful for healing traumatized children. They should be given the opportunity to be involved in positive, nurturing, and caring interactions with peers, teachers, and other caregivers (27, 149). Idsoe et al. (27) accentuate how the many daily hours children spend in schools put educators and school staff in a unique position to support traumatized children. Educators can create trauma-sensitive environments and help traumatized children to feel safe and calm down. If teachers and school staff manage to calm dysregulated children, this will most likely help them with concentration and learning and improve mental health. Educators should also try to identify and be aware of potential triggers children associate with bullying episodes from the past, because if still present, they may elicit fear reactions in bullied children. If necessary, learning environments should be adjusted so that they are better tailored to the needs of the bullied children. However, teachers need to know when they should refer these children to a specialist. This makes it reasonable to assume that treatments must be developed at several tiers so that the interventions can be tailored according to severity. Within schools, three-tier interventions may be a fruitful solution. Then proper interventions can be implemented at the universal tier (for the majority of students), combined with more comprehensive and intensive strategies for students showing moderate problems (selected level), and finally the ones showing high levels of consequences (indicated level). This allows for different combinations of treatments for bullied children and for implementing interventions targeting environmental factors.

## Author Contributions

TI conceptualized and drafted the manuscript and conducted critical revisions. TV, AD, KH, TO, and AN contributed to the conception, drafting of the work, and conducted critical revisions. All authors contributed to the article and approved the submitted version.

## Conflict of Interest

The authors declare that the research was conducted in the absence of any commercial or financial relationships that could be construed as a potential conflict of interest.
